# Agricultural Land Use Determines the Trait Composition of Ground Beetle Communities

**DOI:** 10.1371/journal.pone.0146329

**Published:** 2016-01-05

**Authors:** Helena I. Hanson, Erkki Palmu, Klaus Birkhofer, Henrik G. Smith, Katarina Hedlund

**Affiliations:** 1 Biodiversity, Department of Biology, Lund University, Ecology Building, 223 62, Lund, Sweden; 2 Centre for Environmental and Climate Research, Lund University, Ecology Building, 223 62, Lund, Sweden; Universidade de São paulo, BRAZIL

## Abstract

In order to improve biological control of agricultural pests, it is fundamental to understand which factors influence the composition of natural enemies in agricultural landscapes. In this study, we aimed to understand how agricultural land use affects a number of different traits in ground beetle communities to better predict potential consequences of land-use change for ecosystem functioning. We studied ground beetles in fields with different agricultural land use ranging from frequently managed sugar beet fields, winter wheat fields to less intensively managed grasslands. The ground beetles were collected in emergence tents that catch individuals overwintering locally in different life stages and with pitfall traps that catch individuals that could have a local origin or may have dispersed into the field. Community weighted mean values for ground beetle traits such as body size, flight ability and feeding preference were estimated for each land-use type and sampling method. In fields with high land-use intensity the average body length of emerging ground beetle communities was lower than in the grasslands while the average body length of actively moving communities did not differ between the land-use types. The proportion of ground beetles with good flight ability or a carnivorous diet was higher in the crop fields as compared to the grasslands. Our study highlights that increasing management intensity reduces the average body size of emerging ground beetles and the proportion of mixed feeders. Our results also suggest that the dispersal ability of ground beetles enables them to compensate for local management intensities.

## Introduction

To improve the potential of biological control of agricultural pests, it is fundamental to understand which factors influence the composition of natural enemies in agricultural landscapes [1, 2]. Ground beetle species (Carabidae) are abundant predators on agricultural pests such as cereal aphids [3, 4] and to optimize the contribution of ground beetles to pest control it is essential to identify drivers that shape their community composition [5–7]. Agricultural land-use intensity can influence ground beetle species composition [8–10] as land use affects how suitable habitats are in terms of foraging, reproduction and overwintering [11].

How ground beetles react to land-use changes and how this shapes communities will depend on a number of properties in different species that are described as traits [12, 13]. Studies focusing on the relationship between ground beetle communities and agricultural intensification can thus be informed by existing knowledge about traits in ground beetle species, which may help linking changes in trait composition to expected changes in ecosystem functioning [14–18]. The traits may explain how not only local land use but also the landscape may be important in shaping communities as ground beetle species can disperse considerable distances either by flight [19] or cursorial movement [20, 21]. Thus here we aim to study the trait composition of ground beetle communities that either emerge locally in the fields or actively move across fields. Communities of actively moving species may thus include not only species of local emergence but also those that reproduce and forage in habitats other than those used for overwintering [22, 23].

Increasing land-use intensity is known to benefit carnivorous ground beetles [24, 25], while herbivorous ground beetle species often prefer less intensively managed habitats, such as field margins with grass [7, 26]. Further, increased land-use intensity reduces the average body size of individuals in ground beetle communities [12, 27], which can be of significance for pest control services as the predation rates can be negatively correlated to the average body size of the community [18]. Moreover, frequent agricultural disturbance generally reduces the average body size of soil arthropod communities [28] and it has been suggested that soil tillage can have a negative effect on large ground beetle species [29]. It is generally assumed that adult ground beetles overwinter in grasslands, while larval stages mainly overwinter in arable soils [30, 31]. Differences among species concerning overwintering can have fundamental effects on community characteristics when comparing intensively managed crops with less intensively managed grasslands. Ground beetle species have different moisture preferences [11] and their emergence can be related to soil moisture levels during the overwintering period [32]. Soil moisture content is generally higher when agricultural land use is less intensive as in grasslands [33] suggesting a higher proportion of moisture preferring (hygrophilous) species in those habitats.

Most ground beetles species have flight ability [11], even though many species primarily take flight only for occasional changes of habitat [34, 35]. Generally, ground beetles inhabiting unstable environments are suggested to have good dispersal abilities, facilitating movement to more stable habitats when local conditions become unsuitable [12, 21, 27]. Movement can also be related to trophic level, as predators and parasitoids due to the mobility of their prey may need to move across larger distances as compared to herbivorous species [7, 36]. For cursorial moving ground beetles it has been indicated that the movement range of ground beetles is roughly proportional to their body length [37], suggesting that small species with low ability of flight have a restricted dispersal range. Hence, changes in composition of ground beetle communities in response to agricultural land use are likely to be dependent on dispersal abilities of the species.

In this study we aim to understand how agricultural land use influences ground beetle community composition by using traits related to dispersal and habitat preferences. In order to disentangle effects of the local land use and the possibility of dispersal between fields, ground beetles were sampled using two different methods. Unfenced pitfall traps estimated the composition of actively moving ground beetles which is the result of both species activity and abundance in a field [38] and emergence tents estimated the local emergence of overwintering ground beetles. The ground beetles were collected in three agricultural land-use types ranging from frequently managed sugar beet fields, winter wheat fields to less frequently managed grasslands in an agricultural region of southernmost Sweden. Hence, by comparing the composition of traits of the beetle communities between the two sampling methods in the different types of fields, we determined the key traits that are affected by local land use. We expected that the land use and sampling method would explain the trait distribution in ground beetle communities such as: (1) A lower average body length in more intensively managed crop fields as compared to grasslands since large ground beetles will be negatively affected by disturbances; (2) A higher average body length in actively moving communities as compared to emerging ones since larger ground beetles have a greater cursorial movement rate and will thus dominate catches of unfenced pitfall traps; (3) A higher proportion of species with better flight ability in ground beetle communities in more intensively managed land-use types as species inhabiting these habitats need a better dispersal ability in order to persist; (4) A shift to more carnivore dominated communities in crop fields compared to grasslands reflecting the distribution of resources; (5) A higher proportion of adult hibernators in grasslands, as these are expected to be less common in crop fields; (6) A higher proportion of hygrophilous individuals in grasslands as those soils have a higher water holding capacity.

## Materials and Methods

### Study design

The field work was carried out during April-July 2011 in an agricultural area covering approximately 850 km^2^ located in the province of Scania in south-western Sweden. We selected 22 locations across the study area, with each location comprising three common land-use types: sugar beet, winter wheat and grassland; in total 66 sites. To select study sites and calculate the proportion of arable land at 1000 m radius surrounding each study site, data from the Swedish IACS (Integrated Administration and Control System) database supplied by the Swedish Board of Agriculture was used in ArcGIS software (ESRI). The landscape surrounding each site in year 2010 was dominated by annually tilled crops (77 ± 1%, mean ± SE). The distance between the neighboring locations was approximately 5160 ± 292 m and distances between sites within locations were 682 ± 48 m between grasslands and crop fields and 297 ± 51 m between sugar beet fields and winter wheat fields. The three agricultural land-use types were chosen to represent different levels of management intensities. As our grasslands were not fertilized or treated with pesticides nor tilled on an annual basis, we classified this land-use type as having the lowest management intensity. In contrast both winter wheat and sugar beet fields were intensively managed in terms of tillage, fertilizers and pesticides with sugar beet fields being most recently tilled. All winter wheat fields were conventionally tilled using moldboard plows during autumn when preparing the soil for seed establishment. The sugar beet fields were conventionally tilled in autumn and a second time in spring when preparing the soil for seed establishment. The sampled grasslands included different types of grasslands established on arable land, excluding meadows and grazed semi-natural grasslands. The grasslands were classified according to the Swedish board of agriculture; certified leys (rotational grasslands, n = 9), non-certified leys (rotational grasslands, n = 7), buffer strips (n = 3) and fallows (n = 3). Certified leys should, according to the Swedish board of agriculture, be covered with ley grasses (e.g. *Phleum pratense* L., *Lolium perenne* L.) and/or leguminous plants (e.g. *Trifolium pratense* L., *Medicago sativa* L.) and be cut or grazed on an annual basis. Non-certified leys, buffer strips and fallows can be covered with a more heterogeneous mix of grasses and herbs and may not be cut or grazed on an annual basis. Mean (± SE) field size was 1.7 (± 0.5) ha for grasslands, 16 (± 2) ha for winter wheat and 18 (± 4) ha for sugar beet. The smaller sizes of the grasslands were due to the limited availability of large grasslands in the intensively managed landscapes of the study region. For an overview of the study area see Palmu, Ekroos [9].

### Ground beetle sampling

The activity density [38] of actively moving ground beetles was measured using a single pitfall trap (unfenced) outside the emergence tent at each site. Emerging ground beetles were captured with a pitfall trap within a single emergence tent that was kept at the same position during the whole sampling period in order to obtain a measure of the total productivity of the sampled field [32]. Specimens from pitfall traps were collected at each site (one grassland, one winter wheat and one sugar beet field respectively), giving a total of three emergence tents and three unfenced pitfall traps at each of the 22 locations. In winter wheat and sugar beet fields the traps were located approximately 30 meters from the field edge and in the grasslands around two meters from the edge, due to the smaller sizes of the grasslands. The emergence tents and the unfenced pitfall traps were approximately two meters apart. The tent had a base area of 0.6 × 0.6 meters, a height of 0.6 meters and a white synthetic fabric with, 16.7 × 5 mesh cm^-2^ (MegaView Science^™^). To avoid ground beetles escaping or entering the tent, flaps around the base of the tent were inserted into the soil down to a depth of 10 cm. During the study period, the vegetation inside the tents was regularly cut to approximately a third of the tent height, and the plant cuttings were removed. Each pitfall trap consisted of a plastic cup (9 cm diameter, 6 cm height) with a 15 × 15 cm metal sheet roof placed about 2 cm above the unfenced pitfall trap. The plastic cups, both inside and outside the tent, were dug down into the soil so that the rim was on the same level as the soil surface. The cups were partially filled with 50–70% propylene glycol and a small amount of detergent. All traps were emptied and ground beetles were collected every fortnight from the end of April until the end of July. The ground beetles were transferred to ethanol (70%) for storage. Due to a very high number of ground beetles in the unfenced traps, we only analysed samples from the second sampling period each month.

### Ground beetle trait classification

The ground beetles were classified to species level according to Lindroth and Bangsholt [39] and Luff [40] with *Amara spp*., *Harpalus spp*. (except *Harpalus rufipes* Degeer) and *Ophonus spp*. only being classified to genus. The trait categorization on each species identified within the study was based on information from Lindroth and Bangsholt [39], Lindroth [35] and the public database http://carabids.org [41] and some additional literature (see S1 Table). We calculated Community-Weighted Means (CWMs) for four categorical traits (flight ability, feeding preference, overwintering state and moisture preference) and one continuous trait (body length) to describe changes in trait composition [29, 42, 43] depending on land-use type and sampling method. CWMs for ground beetles were calculated using the following species coding: (i) Flight ability (poor = -1 or good = 1): species with poor flight ability included those with no or reduced wings, dimorphic species and those for which flight observations are missing or rare, while species with good flight ability included those with fully developed wings and those for which flight has been documented; (ii) Feeding preference (mixed feeders = -1 or carnivorous = 1): species with mixed feeding habits included those that are omnivorous/herbivorous, while carnivorous species included those with mainly carnivorous diet; (iii) Overwintering state (larval = -1 or adult = 1); (iv) Moisture preference (xerophilic = -1, heterophilic = 0 or hygrophilic = 1); (v) Body length (3–24 mm).

### Statistical analysis

A total number of 7544 individual of 44 species was used for calculating CWMs of the traits (see S1 Table). Prior to statistical analysis the data sets of each site and method were pooled over the season and the abundance data was used to calculate CWM traits and the resemblance matrix for species composition. For each trait, CWMs were calculated by summing the products of the species trait and the corresponding species abundance and finally dividing the sum of the products by the total ground beetle abundance. We calculated correlation coefficients between traits to determine if they could be treated independently. CWMs of the traits flight ability and diet (Pearson r = -0.59) had the strongest observed correlation coefficients and we assume that each individual trait therefore contained enough independent information to justify further analyses. Due to a few missing samples on some sampling dates a number of sites were removed before the statistical analysis, causing a slightly unbalanced design compared to the originally set of 22 sites from each land-use type (see S1 Table). Species composition was analyzed based on untransformed abundance values and Bray-Curtis similarity.

Permutational analysis of variance (PERMANOVA), [44] was used to test for the factors and their interactions on overall CWM trait composition and species composition of ground beetle communities, with the random factor “location” and the fixed factors “land-use type” and “sampling method”. A multivariate model was used to identify model terms for follow-up tests of individual CWM traits and to thereby protect our tests against multiple testing e.g. protected ANOVA approach, [45]. Only for this model CWM trait values were scaled (mean = 0; sd = 1) and the standardized trait CWMs were then used to calculate a resemblance matrix based on Euclidean distances. All p-values are derived from 9999 permutations of the residuals under a reduced PERMANOVA model [44]. The following models for individual traits were then only performed for model terms that were significant in the multivariate global test. For tests of individual trait variables, standardization across traits was not needed and raw CWMs were used. All other model specifications are identical to the multivariate model. PERMANOVA post-hoc tests were performed to compare individual levels of the factor “land-use type” in cases where the factor was significant in the main model. Henceforth when we use the term “trait composition” or the individual CWM trait names we refer to the community-weighted means for the traits. The effect of land-use type and method on species composition is illustrated using a Canonical analysis of principle coordinates (CAP, [46]) with a combined land-use type x method factor to constrain the ordination due to the significant interaction between the two main factors in the PERMANOVA model. To relate the resemblance matrices for species and trait composition we used a non-parametric version of the Mantel test based on Spearman rank order correlations. All statistical analyses were performed using the software Primer-E v6 [47] and the PERMANOVA+ add-on [48].

### Ethics Statement

The field study did not involve endangered or protected species. All sampling took place on private land with permission from the land owners to conduct fieldwork. Ethical approval was not required as we worked with arthropods.

## Results

**Fig 1:**
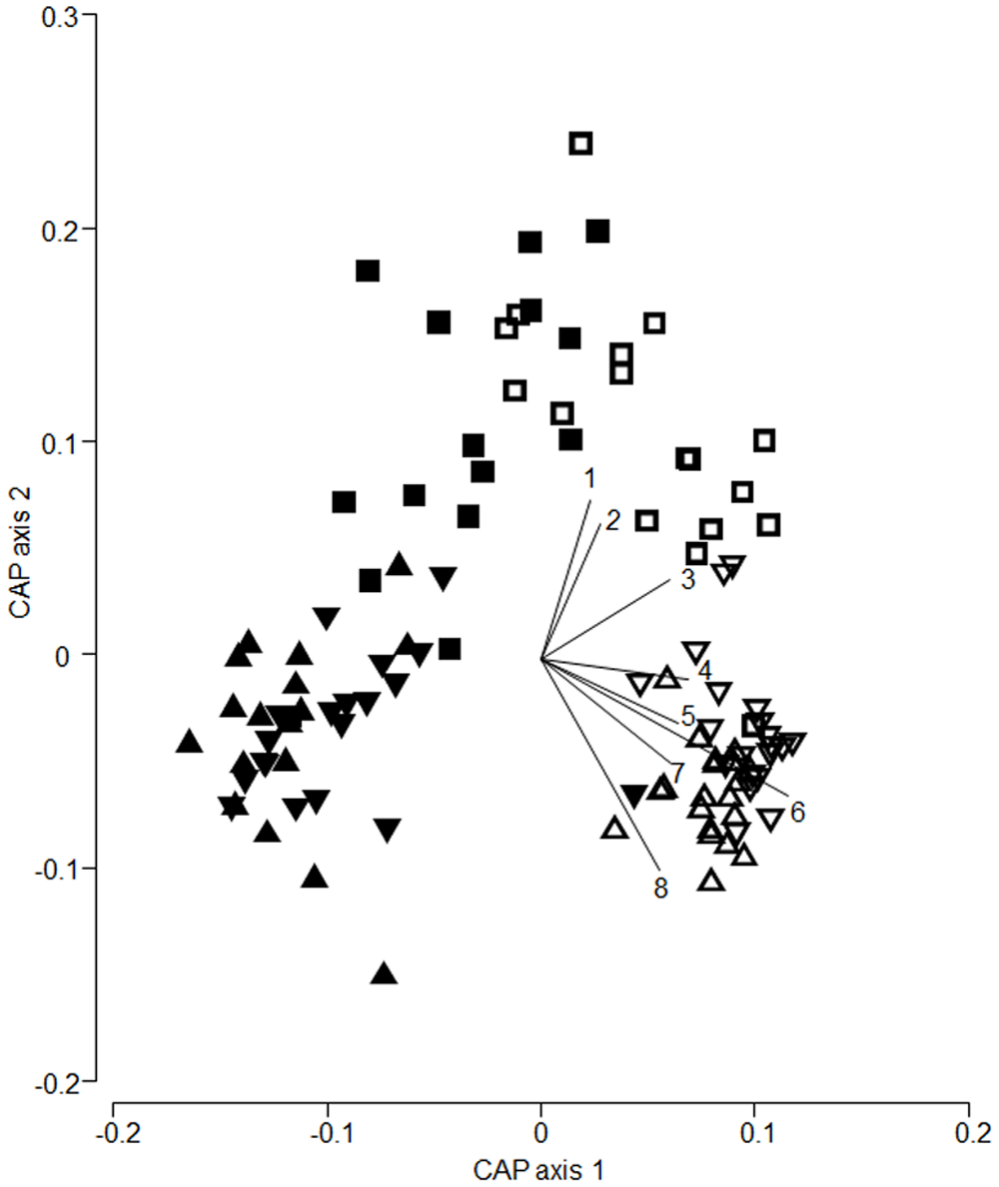
Canonical analysis of principle coordinates (CAP) based on untransformed abundances of ground beetles species and Bray-Curtis similarities between sites and a joint factor for method (pitfall traps outside tents are represented by open symbols and pitfall traps in emergence tents are represented by closed symbols) and land use (grasslands (■), wheat (▼) and sugar beet fields (▲)). Vectors are superimposed for species with a Pearson correlation coefficient > 0.4. Species are 1, *Poecilus versicolor*; 2, *Amara* spp.; 3, *Harpalus rufipes*; 4, *Pterostichus niger*; 5, *Anchomenus dorsalis*; 6, *Pterostichus melanarius*; 7, *Bembidion lampros*; 8, *B*. *obtusum*.

The species composition of ground beetle communities differed significantly between the land-use types (*F*_2,107_ = 9.43, *P* < 0.001) and sampling methods (*F*_1,107_ = 43.10, *P* < 0.001) with a significant interaction between both factors (*F*_2,107_ = 3.56, *P* < 0.001). Three species (*Poecilus versicolor*, *Harpalus rufipes* and *Amara spp*.) were more common in grasslands, whereas *Pterostichus melanarius* and *Bembidion obtusum* were most abundant in communities of actively moving ground beetles in crop field (species vectors in Fig 1). Sites that were related according to their species composition also resembled each other based on trait composition (Mantel test, *ρ* = 0.72, *P* < 0.001), reflecting that differences in community composition between sites were characterized by differences in trait and species composition.

**Fig 2:**
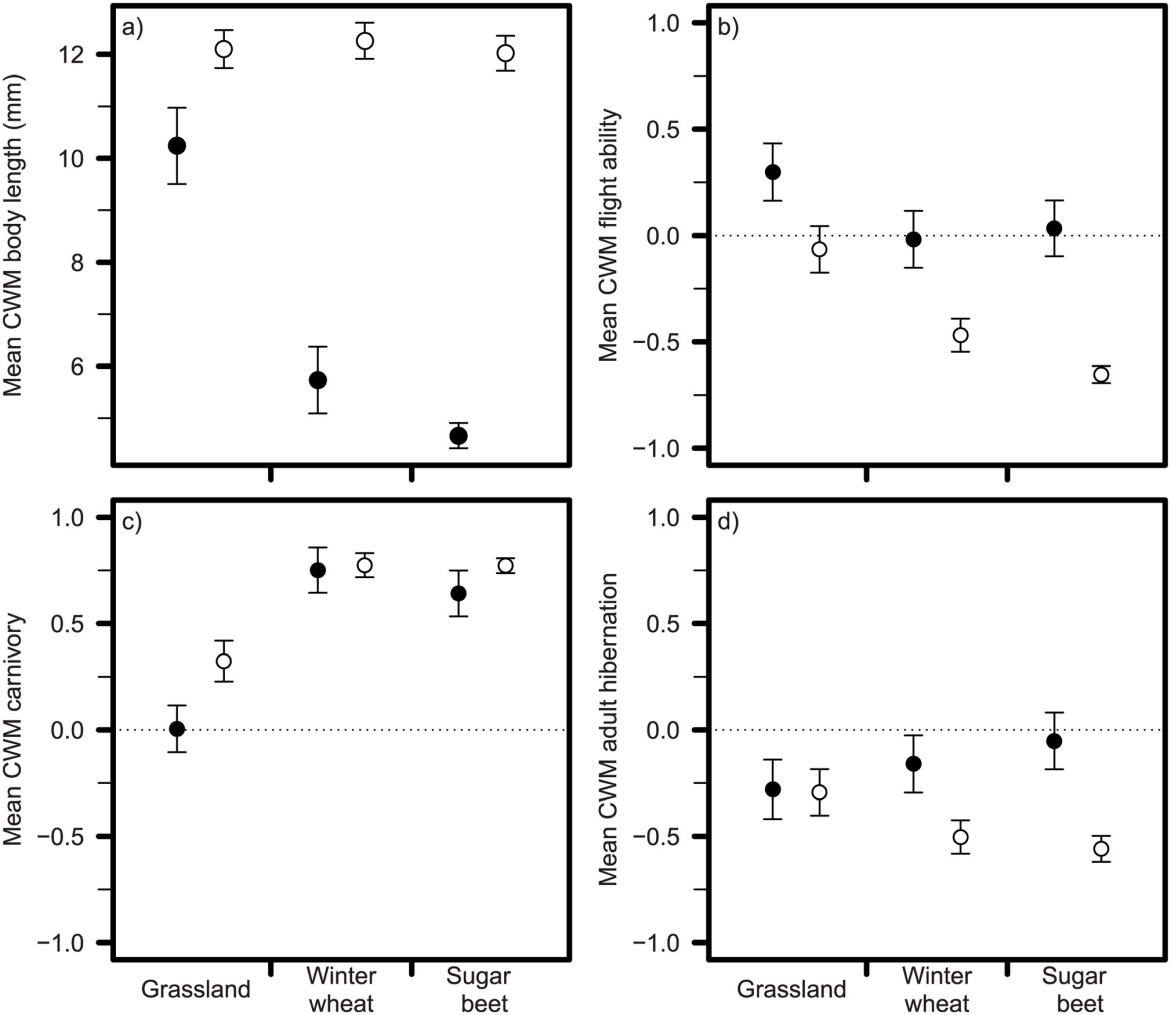
Community-weighted means (CWM ± SE) for traits: a) body length, b) flight ability, c) carnivory and d) adult overwintering in the three agricultural land-use types: grasslands, winter wheat and sugar beet fields for emerging (●) and actively moving (○) ground beetle. Note that a value of “1” (in panel b, c and d) indicates that all individuals in a community had good flight ability (panel b), were carnivorous (panel c) or overwintered as adults (panel d). Vice versa, a value of “-1” indicates that all individuals in a community had poor flight ability (panel b), were herbivorous/omnivorous (panel c) or overwintered as larvae (panel d). The broken horizontal line at the value zero of the y-axis in panel b, c and d indicate the point where the proportion of the opposed traits are equal.

The CWM trait composition of ground beetle communities differed significantly between the land-use types (*F*_2,107_ = 9.54, *P* < 0.001) and sampling methods (*F*_1,107_ = 30.89, *P* < 0.001) with a significant interaction between both factors (*F*_2,107_ = 3.30, *P* = 0.002). An increasing land-use intensity was related to a lower CWM body size in emerging communities but not in actively moving ones (*F*_2,107_ = 20.23, *P* < 0.001; Fig 2a). There was a higher CWM flight ability in grasslands compared to crop fields (*F*_2,107_ = 10.56, *P* < 0.001; Fig 2b) and in emerging as compared to actively moving communities (*F*_1,107_ = 48.08, *P* < 0.001; Fig 2b). The proportion of carnivorous individuals (CWM carnivory) was significantly higher in crop field compared to grassland communities (*F*_2,107_ = 33.94, *P* = 0.001; Fig 2c) and in actively moving compared to emerging communities (*F*_1,107_ = 7.25, *P* = 0.006; Fig 2c). Species that overwinter as adults (CWM adult hibernation) were proportionally more abundant in emerging communities as compared to active ones (*F*_1,107_ = 11.90, *P* = 0.003; Fig 2d), but did not differ significantly between land-use types. CWM moisture preference did not differ significantly among land-use types or sampling methods.

## Discussion

This study shows that ground beetle trait composition is affected by agricultural land use, and can also differ between communities of emerging and actively moving ground beetles. In partial agreement with our hypothesis, the average body length of emerging ground beetles was lower in communities of crop fields than in grasslands. Previous studies across a range of habitats from coniferous forest to crop fields documented that the average body length of actively moving ground beetles and the proportion of species that are capable of flight were reduced by an increase in land-use intensity [12, 27]. However, within an agricultural context such patterns do not seem to be valid for the actively moving ground beetles as we show that ground beetles with poor flight ability and with a large body size dominated the communities in crop fields. Thus, our study highlights that patterns of ground beetle trait composition observed previously in land-use gradients across natural, semi-natural and agricultural land may not be generalized in the context of managed land-use types of agricultural landscapes.

The larger difference in species composition between emerging and actively moving communities in crop field as compared to grasslands was mainly driven by higher abundance of *Pterostichus melanarius* and *Bembidion obtusum* in communities of actively moving beetles in crop field as compared to communities of emerging individuals. These two species are generally associated to intensively managed agricultural land [11, 39, 41]. Three other species (*Poecilus versicolor*, *Harpalus rufipes* and *Amara spp*.) were more abundant in grasslands as compared to crop fields. All three are arguably species/genera with closer association to less intensively managed grasslands as compared to crop fields [11, 39, 41]. Although the resemblance between sites based on species composition was similar to the resemblance between sites based on CWM trait composition, we found that the CWM trait based analysis (discussed below) revealed information that could not have been disclosed by solely focusing on species composition analysis.

It has previously been shown that frequent agricultural disturbances lead to lower average body length of soil communities spanning multiple invertebrate taxa [28]. Here we show for the first time that this can be a general pattern also for aboveground arthropods that overwinter in soil. There are several mechanisms that can explain why the average body length of emerging ground beetles is lower at increasing land-use intensity. The mortality due to physical disturbances such as tillage may be higher among larger ground beetles as compared to smaller ones [29]. However, large ground beetles may also migrate to less disturbed overwintering sites more frequently [23], as they have higher cursorial movement rates compared to smaller species [37, 49]. Furthermore it has been suggested that intraguild predation may reduce emergence of small ground beetles in boundary habitats as compared to crop fields [50]. In contrast to previous studies [12, 27] and our own expectation the average body length of actively moving communities did not depend on the land-use intensity. This is probably due to the different range of ecosystems covered in the different studies. Ribera, Doledec [12] and Blake, Foster [27] covered gradients of spatially separated landscapes from forest to farmland where species pools are markedly different between habitats. In contrast, the different land-use types in our study share a similar species pool to a large extent and the most dominant species in all land use types was the relatively large species *Pterostichus melanarius* which is known to dominate ground beetle communities in agricultural landscapes [7, 25]. In general the average body length was higher in communities of actively moving ground beetles as compared to those of emerging ones. This difference in average body length is probably a combined result of lower emergence and higher cursorial movement rates by larger species [37, 49].

In contrast to our hypothesis, grasslands had higher proportion of ground beetles with good flight ability as compared to winter wheat and sugar beet fields. This result may at first appear to contradict previous findings by Ribera, Doledec [12] reporting a higher frequency of macropterous species with increasing land-use intensity. However, in our study area the grasslands were not only the least intensively managed habitat but also generally scarce and isolated which may have influenced the community trait composition concerning dispersal ability. Dispersal is generally expected to have a strong impact on the likelihood of survival in fragmented habitats [51, 52] and Hendrickx, Maelfait [53] showed that the number of ground beetle species with good flight ability increased with increasing distances to other semi-natural habitat patches. Moreover de Vries, den Boer [54] found that heathland ground beetle species with a poor flight ability were more affected by fragmentation than species with a high flight ability. Other recent studies have also demonstrated that species with poor flight ability generally dominate ground beetle communities in crop fields [29] and are unaffected by increasing management intensity [16]. Thus, good flight ability does not seem to be the main driver for persistence in crop fields, but can be important for survival in marginalized habitats in intensively managed agricultural landscapes. The flight ability was lower among actively moving ground beetles as compared to emerging ones, which could be explained by the dominance of the two generally brachypterous and flightless species *P*. *melanarius* and *B*. *obtusum* in the actively moving ground beetle communities.

Carnivorous ground beetles were more common in communities in intensively managed crop field than in grasslands, which is in line with other studies showing that arthropods with a carnivorous diet can benefit from increasing management intensity [16, 24]. The widespread species *P*. *melanarius* dominated ground beetle communities in our study area which agrees with previous studies in agricultural landscapes with high proportions of arable land [7, 25]. The contrast of a high proportion of carnivorous ground beetles in crop fields and a greater proportion of herbivores and omnivores in grasslands is probably due to differences in the availability of food resources e.g. [55]. Grasslands have a higher plant species diversity and availability of plant-derived food resources than crop fields [56], thus benefitting herbivorous ground beetle species [26]. Furthermore, arthropod pest species as e.g. aphids can be abundant food resources and attract ground beetle species to crop fields [57, 58]. Thus, carnivorous ground beetles are not only benefitting from management intensification within a single crop type [26], but also from more intense management across different agricultural land-use types.

In disagreement to our hypothesis concerning overwintering traits, we did not find that the land-use type affected the proportion of adult hibernators. Instead our results showed that the proportion of adult hibernators was higher in communities of emerging ground beetles as compared to actively moving ones. Thus our results do not support the general assumption that emergence of ground beetles that overwinter as larva are more associated to crop fields than to field boundaries [30]. Larval and egg stages can be more vulnerable to disturbance e.g. tillage, as compared to adults [59] and even though hibernating larvae may be more common in soils of crop fields they may suffer high mortality due to soil management. The higher proportion of larval hibernators among the actively moving ground beetles as compared to the emerging ones can be explained by the cursorial dispersal of the dominant larval overwintering species *P*. *melanarius*. In contrast to our expectations, hygrophilous ground beetles were not more common in grasslands as compared to crop fields. Thus, even though soil water holding capacity is generally higher in grasslands in the study region [60], it did not lead to a higher proportion of hygrophilous ground beetles. This indicates that other traits are more important when explaining ground beetle distribution across different agricultural land-use types.

In conclusion, our results show that increasing management intensity reduces the average body size of emerging ground beetles and the proportion of mixed feeders. This, in combination with recent results imply that predation rates can be higher in generalist predator communities with individuals of smaller average body size [18] and suggests that increasing land-use intensity may in fact promote the biological control potential of ground beetles. Our results suggest that the dispersal ability of ground beetles enables them to compensate for local management intensities. To predict this dynamic in more detail in order to provide management advice further studies are needed. Here we show that grasslands might be of lower importance for the biological control potential by ground beetles. However grasslands are still valuable for the conservation of other ground beetle traits and ecosystem services delivered by arthropods in general [61–63].

## Supporting Information

S1 Table Ground beetle species trait codes: Body length (BL) (mm), major diet (MD) (omnivorous/ herbivorous = -1, carnivorous = 1), flight ability (FA) (poor = -1, good = 1), major overwintering development stage (OS) (larvae = -1, adult = 1), moisture preference (MP) (xerophilic = -1, heterophilic = 0, hygrophilic = 1). Total abundances of all ground beetle species used in the analysis displayed separately in emergence tents (ET) and unfenced pitfall traps (UP) across the grasslands, winter wheat fields and sugar beet fields sampled in year 2011.
(PDF)
