# Supplementary material for: Agricultural Land Use Determines the Trait Composition of Ground Beetle Communities
Source: PLoS One. 2016 Jan 5;11(1):e0146329. doi: 10.1371/journal.pone.0146329 (PMC4711665; doi:10.1371/journal.pone.0146329)
Supplement: S1 Table — Total abundances of all ground beetle species used in the analysis displayed separately in emergence tents (ET) and unfenced pitfall traps (UP) across the grasslands, winter wheat fields and sugar beet fields sampled in year 2011. (PDF) [file pone.0146329.s001.pdf]

Supporting information 1

Agricultural land use determines trait composition of ground beetle communities.

Helena I. Hanson [1, 2*], *Erkki Palmu* [1,*], *Klaus Birkhofer* [1], *Henrik G. Smith* [1,2], and *Katarina Hedlund* [1,2]

Ground beetle species trait codes: Body length (BL) (mm), major diet (MD) (omnivorous/ herbivorous =-1, carnivorous=1), flight ability (FA) (poor=-1, good=1), major overwintering development stage (OS) (larvae=-1, adult=1), moisture preference (MP) (xerophilic=-1, heterophilic=0, hygrophilic=1). Total abundances of all ground beetle species used in the analysis displayed separately in emergence tents (ET) and unfenced pitfall traps (UP) across the grasslands, winter wheat fields and sugar beet fields sampled in year 2011.

Species						Grassland		Winter wheat		Sugar beet	
**Name**	**FA**	**OS**	**MD**	**MP**	**BL**	**ET** n=15	**UP** n=17	**ET** n=19	**UP** n=20	**ET** n=17	**UP** n=20
Acupalpus meridianus (L. 1761)	1	1	-1	1	3.6	0	0	0	0	1	1
Agonum muelleri (Herbst 1784)	1	1	1	0	8.3	0	0	0	13	0	2
Amara spp. (Bonelli 1810)	1	1	-1	-1	7.5	27	137	0	6	1	4
Anchomenus dorsalis (Pontoppidan 1763)	1	1	1	0	7.1	1	12	4	177	0	22
Badister bullatus (Schrank 1798)	1	1	1	0	5.7	1	6	0	0	0	0
Bembidion aeneum (Germar 1824)	-1	1	1	1	4	0	0	0	0	0	1
Bembidion gilvipes (Sturm 1825)	-1	1	1	1	2.8	0	1	0	0	0	0
Bembidion guttula (F. 1792)	1	1	1	1	3.2	0	0	1	0	0	0
Bembidion lampros (Herbst 1784)	-1	1	1	0	3.7	2	9	5	27	4	127
Bembidion mannerheimii (Sahlberg C.R. 1827)	-1	1	1	1	3.1	0	1	0	0	0	0
Bembidion obtusum (Audinet-Serville 1821)	-1	1	1	0	3.2	5	12	50	111	53	189
Bembidion properans (Stephens 1828)	-1	1	1	1	3.9	0	1	4	2	0	0
Bembidion quadrimaculatum (L. 1761)	1	1	1	1	3.2	0	2	2	3	2	4
Bembidion tetracolum (L. 1758)	-1	1	1	1	5.5	3	3	11	19	9	7
Blemus discus (F. 1792)	1	-1	1	1	5	0	4	0	0	0	0

Calathus erratus (Sahlberg C.R. 1827)	-1	-1	1	-1	10.2	0	0	0	1	0	6
Calathus fuscipes (Goeze 1777)	-1	-1	1	-1	12.2	3	6	0	16	0	7
Calathus melanocephalus (L. 1758)	-1	-1	1	-1	7.4	2	0	1	6	0	3
Carabus granulatus (L. 1758)	-1	1	1	1	20	0	2	0	1	0	0
Carabus nemoralis (Müller O.F. 1764)	-1	1	1	0	24	0	1	0	0	0	0
Clivina fossor (L. 1758)	1	1	-1	1	6	16	24	8	23	26	86
Demetrias atricapillus (L. 1758)	1	1	1	0	5.1	2	0	1	14	1	1
Harpalus rufipes (De Geer 1774)	1	-1	-1	0	13.4	83	130	6	109	4	117
Harpalus spp.(Latreille 1802)	1	1	-1	0	10	22	23	8	26	2	76
Leistus ferrugineus (L. 1758)	-1	-1	1	0	7.3	2	0	0	0	0	0
Loricera pilicornis (F. 1775)	1	1	1	1	7.3	0	5	0	23	0	1
Nebria brevicollis (F. 1792)	1	-1	1	1	12	4	3	0	6	0	4
Notiophilus aesthuans (Motschulsky 1864)	1	1	1	-1	5.3	1	1	3	8	0	9
Notiophilus germinyi (Fauvel 1863)	-1	-1	1	-1	6	0	0	0	2	0	0
Ophonus spp. (Stephens 1828)	1	1	-1	-1	8	20	0	0	1	0	0
Paradromius linearis (Olivier 1795)	-1	1	1	-1	5.2	0	1	0	0	0	0
Poecilus cupreus (L. 1758)	1	1	-1	1	12.2	5	22	1	104	0	15
Poecilus lepidus (Leske 1785)	-1	-1	1	-1	13.1	0	0	0	2	0	0
Poecilus versicolor (Sturm 1824)	1	1	1	0	10.6	4	55	0	1	0	1
Pterostichus melanarius (Illiger 1798)	-1	-1	1	0	15	65	579	54	1720	7	2079
Pterostichus niger (Shaller 1783)	-1	-1	1	1	17.8	8	38	0	62	0	26
Pterostichus strenuus (Panzer 1796)	-1	1	1	1	6.6	0	1	0	0	0	0
Pterostichus vernalis (Panzer 1795)	-1	1	1	1	6.8	0	1	0	0	0	0
Syntomus foveatus (Geoffroy in Fourcroy 1785)	-1	1	1	-1	3.5	0	2	0	0	0	0
Syntomus truncatellus (L. 1761)	-1	1	1	-1	2.9	0	1	0	0	0	0
Synuchus vivalis(Illiger 1798)	-1	1	-1	-1	7.3	2	2	3	8	0	22
Trechoblemus micros (Herbst 1784)	1	1	1	1	4.3	0	3	0	2	0	3
Trechus quadristriatus (Schrank 1781)	1	-1	1	-1	3.8	16	12	68	134	52	142
Trechus secalis (Paykull 1790)	-1	-1	1	1	3.8	11	15	31	41	32	46
**Sum**						305	1115	261	2668	194	3001

Hendrickx F et al. (2009) Pervasive effects of dispersal limitation on within- and among-community species richness in agricultural landscapes. Global Ecol. Biogeogr. 18:607-616. doi: DOI 10.1111/j.1466-8238.2009.00473.x

Homburg K, Homburg N, Schäfer F, Schuldt A, Assmann T (2013) Carabid.org- A dynamic database of ground bettle traits (Coleoptera, Carabidae). Insect Conserv. Divers. 7:195–205. doi: 10.1111/icad.12045

Koch, K. 1989. Die Käfer Mitteleuropas, Bd. E1: Carabidae-Micropeplidae. Spektrum Akademischer Verlag, Heidelberg, Germany.

Lindroth CH, Bangsholt F (1985) The Carabidae (Coleoptera) of Fennoscandia and Denmark. Brill, Leiden, Netherlands

Lindroth CH (1992) Ground Beetles (Carabidae) of Fennoscandia: A Zoogeographic Study: Part 1. Specific knowledge regarding the species. Intercept Ltd, Andover, UK

Luff ML (2007) The Carabidae (ground beetles) of Britain and Ireland, 2nd edn. Royal Entomological Society, St. Albans, UK

Ribera I, Doledec S, Downie IS, Foster GN (2001) Effect of land disturbance and stress on species traits of ground beetle assemblages. Ecology 82:1112-1129. doi: 10.1890/0012-9658(2001)082[1112:eoldas]2.0.co;2
